# Immune Dysregulation and Cytokine Profiling in Acute *Mycoplasma pneumoniae* Pneumonia

**DOI:** 10.3390/microorganisms14010229

**Published:** 2026-01-19

**Authors:** Ying Wen, Yanfang Zhai, Shuli Sang, Chen Cao, Yunyun Mao, Enbo Hu, Lina Zhai, Xuanqi Ye, Kai Li, Yanchun Wang, Rui Yu

**Affiliations:** 1 School of Pharmacy, Nanjing University of Chinese Medicine, Nanjing 210023, China; wy2811356919@163.com; 2 Academy of Military Medical Sciences, Beijing 100071, China; yfzhai0704@163.com (Y.Z.); sslsandra@126.com (S.S.); caoch24biotech@126.com (C.C.); myy-0706@163.com (Y.M.); enbohu180466@163.com (E.H.); zhailina8023@163.com (L.Z.); stpun23@163.com (X.Y.); 13910096703@139.com (K.L.); 3 College of Chemistry and Life Science, Beijing University of Technology, Beijing 100124, China

**Keywords:** *Mycoplasma pneumoniae*, immunity, cytokines, inflammation, lung injury

## Abstract

*Mycoplasma pneumoniae* pneumonia (MPP) is a common respiratory infection characterized by significant inflammatory responses and lung tissue injury. However, the precise immunological mechanisms and temporal dynamics of key cytokines driving pulmonary inflammation in MPP are still unclear. This study aimed to investigate the underlying immunological mechanisms and cytokine dynamics in MPP. We established an acute MPP murine model via intranasal administration of *M. pneumoniae*. This model recapitulates key features of human MPP, such as robust airway inflammation and cytokine production. Comprehensive analyses were conducted, including histopathology, flow cytometry, and cytokine profiling. Results showed severe inflammatory responses with prominent infiltration of neutrophils and macrophages in lung tissue, whereas monocyte populations were significantly reduced, indicating a shift towards myeloid cell predominance. Notably, 36 cytokines, including pro-inflammatory interleukins (IL-1β, IL-6, IL-17A) and chemokines, were statistically significantly upregulated in bronchoalveolar lavage fluid compared to the normal group, highlighting a cytokine storm associated with lung inflammation and tissue damage. Gene Ontology (GO) and Kyoto Encyclopedia of Genes and Genomes (KEGG) signaling pathway analysis further revealed enriched pathways related to cytokine-cytokine receptor interactions and IL-17 signaling, suggesting potential therapeutic targets. In conclusion, this study preclinical provides insights into the innate immune response and cytokine-driven pathology in acute MPP, underscoring the pivotal roles of myeloid cells and pro-inflammatory cytokines. Future research should focus on clinical validation of these findings to assess their translational potential and the exploration of immunomodulatory strategies informed by this model to mitigate MPP severity.

## 1. Introduction

*Mycoplasma pneumoniae* (MP) is an intracellular pathogen responsible for a variety of respiratory conditions in humans, including pneumonia, tracheobronchitis, pharyngitis, and asthma. The microorganism has the capability to invade and persist within host cells, which can trigger an exaggerated immune response [1,2]. The study of MP infection is critical as it represents a significant cause of community-acquired pneumonia, particularly among children and young adults [3,4,5]. Despite its prevalence, the pathogenic mechanisms underlying the immunological responses during acute *Mycoplasma pneumoniae* pneumoniae (MPP) remain inadequately characterized [6]. Severe cases can result in substantial pulmonary damage and extrapulmonary complications [7,8], emphasizing the need for a deeper understanding of the host immune responses involved in MPP pathology.

The network of inflammatory interactions formed by innate and adaptive immunity plays an important role in MPP and is closely related to disease severity [9,10]. Several studies have reported that children with severe MPP (SMPP) have significantly higher levels of white blood cells and inflammatory markers, but lower lymphocytes and natural killer cells than the general MPP group (GMPP) group [11,12,13]. Specific bacterial components, notably CARDS toxin and lipoproteins in MP infection, intensify neutrophil recruitment via signaling pathways, including the IL-23/IL-17 axis and GM-CSF [14,15,16]. The neutrophil-to-lymphocyte ratio can serve as a predictor of poor prognosis in patients with MPP, including the occurrence of necrotizing pneumonia (NP), refractory MPP (RMPP), and other adverse outcomes [17]. Upon MPP infection, alveolar macrophages (AMs) are attracted, activated and subsequently induce cytokine and chemokine responses [18]. The MP lipoprotein binds Toll-like receptors (TLRs) on AMs, activating nuclear factor (NF)-κB and causing proinflammatory cytokine secretion, promoting neutrophil aggregation and pathogen phagocytosis [19,20].

Previous research has identified *Mycoplasma pneumoniae* as a major etiological agent of respiratory infections, and several studies have linked the infection to the production of pro-inflammatory cytokines and the involvement of various immune cells [21,22]. However, the precise mechanisms through which MP elicits severe inflammation and immune dysregulation remain unclear. This gap is critically underscored by the emergence of macrolide-resistant strains, which pose a significant clinical challenge [23,24]. This challenge directly motivates the need for a deeper understanding of host-directed immune responses as an alternative therapeutic avenue. Thus, a comprehensive analysis of the local and systemic immune landscape during MP infection is essential to c immune-based interventions that could complement or circumvent existing antibiotic limitations.

In this study, we focus on the inflammatory cytokine and chemokine networks, as well as the composition of innate immune cells—such as neutrophils, macrophages, dendritic cells, and monocytes—and adaptive immune cells in the lungs during acute MP infection. We integrate multiplex cytokine analyses and high-dimensional mass cytometry (CyTOF) to gain a systems-level understanding of the immune response. This approach moves beyond the analysis of individual cytokines to uncover coordinated signaling pathways that contribute to disease severity. Specifically, the objectives of this study are: (1) to integrate multiplex cytokine profiling and high-dimensional immune cell phenotyping to construct a comprehensive map of the local immune landscape in acute MPP; (2) to identify the key cytokine-driven networks and specific immune cell subsets that correlate with and potentially drive pathological inflammation; (3) to synthesize these findings into a cohesive model of the immunopathogenic mechanisms underlying MPP, with a focus on the interplay between innate and adaptive immunity. This comprehensive approach aims to elucidate the complex interplay of immune factors involved in MPP, laying the foundation for future therapies.

## 2. Materials and Methods

### 2.1. M. pneumoniae Culture and Reagents

The MP FH strain was obtained from the American Type Culture Collection (ATCC 15531, Manassas, VA, USA). This strain was chosen for infection modeling due to its prevalent use in MPP studies and its robust ability to induce lung inflammation in mice [25], aligning with the goals of our study. The macrolide-resistant variant (A2063G) and strain M129 were generously supplied by the Capital Institute of Pediatrics. The MP strain was cultured in a broth medium (Antu, Zhengzhou, China) at 37 °C and 5% CO_2_ in a BSL-2 laboratory until a distinct yellow color change was observed. Quantification of MP during its logarithmic growth phase was performed using color change units (CCU) alongside quantitative real-time PCR (qPCR) as complementary methods. The MP culture was then aliquoted as the initial stock solution and stored at −80 °C for preservation.

### 2.2. Establishment of MPP Mice Model

Specific pathogen-free female BALB/c mice (6–8 weeks of age, weighing 18 ± 2 g) were obtained from Beijing Vital River Laboratory Animal Technology Co., Ltd. (Beijing, China). The mice were maintained in groups of 4 per cage at a controlled environment of 22–26 °C with 40–60% relative humidity under a 12 h light/dark cycle. They were provided with unrestricted access to food and water and housed in an ABSL-2 laboratory. A total of twelve BALB/c mice were selected based on their body weight and then randomly divided into two groups of six each: the normal control group (NC) and the MPP group. The MPP mice received a 50 µL intranasal inoculation of a 1 × 10^9^ CCU/mL MP suspension once daily for three consecutive days. In contrast, the control group received an equivalent volume of saline solution. All experimental procedures were carried out in strict accordance with the protocols set forth by the Laboratory Animal Administration Committee of the Beijing Institute of Biotechnology, ensuring adherence to ethical principles governing animal research.

### 2.3. Hematoxylin and Eosin Staining (HE)

The histopathological examination of the mice lung tissue was conducted using HE staining, a method that highlights cellular and tissue structures. Samples were first immersed in 10% formalin, dehydrated with ethanol, embedded in paraffin, and sectioned at a thickness of 4 μm. The sections were then deparaffinized and stained with hematoxylin and eosin.

### 2.4. Quantitative Real-Time PCR

*Mycoplasma pneumoniae* real-time PCR kit was purchased from Liferiver (Shanghai, China) for the detection of MP DNA in samples. Prior to utilization, the nucleic acid extraction solution should be thoroughly mixed to ensure the uniform distribution of any insoluble particles it contains. The *Mycoplasma pneumoniae* stock solution was serially diluted tenfold before being added to 50 μL of nucleic acid extraction solution. The mixture was incubated in a water bath at 100 °C for 10 min, then centrifuged at 13,000 rpm for 5 min, and 4 μL of the resulting supernatant was used as the PCR template. Take the reagents in volumes proportional to the number of reaction tubes (*n*): 35 μL MP nucleic acid fluorescent PCR detection mixture, 1 μL internal control, and 0.4 μL enzyme (Taq + UNG) per tube. Vortex the mixture for 5 s, then centrifuge at 3000 rpm for 1 min. The real-time PCR was performed in a total volume of 40 μL using the Bio-Rad qPCR system (Richmond, CA, USA). The protocol included an initial incubation at 37 °C for 2 min, denaturation at 94 °C for 2 min, followed by 40 cycles of 93 °C for 15 s and 60 °C for 60 s. Selection of fluorescent channels for detection: the FAM and HEX channels were selected.

### 2.5. Ella^TM^ Microfluidic-Based Platform

The bronchoalveolar lavage fluid (BALF) was collected through three lung lavages with saline via intubation, and serum was taken from anesthetized mice. Both samples were diluted twofold and assayed for four inflammatory mediators (IL-17, IL-6, IL-1β, and TNF-α) using the Simple Plex Cartridge on an Ella™ System (ProteinSimple, San Jose, CA, USA) [26]. The Ella™ System can detect sensitivities from femtograms (fg) to picograms (pg) per milliliter.

### 2.6. BALF Cytokine Multiplex Analysis

Proteins were measured using the Olink Target 48 Cytokine Panel (Olink Proteomics AB, Uppsala, Sweden) according to the manufacturer’s instructions [27]. The Proximity Extension Assay (PEA) technology simultaneously analyzed 45 analytes in 1 μL of sample. A pair of oligonucleotide-labeled antibody probes bound to their target proteins. When the two probes approached each other, the oligonucleotides hybridized. The addition of DNA polymerase initiated proximity-dependent DNA polymerization events, which generated unique PCR target sequences. The resulting DNA sequences were detected and quantified using a microfluidic real-time PCR instrument. The results of the assay are expressed in NPX^®^ values, which are arbitrary units measured on a log2 scale, indicating that higher values reflect increased levels of protein expression. For each protein analyzed across both datasets, assessments of normality and homogeneity of variance were conducted. To evaluate the normality of the distribution of log2-transformed values, the Shapiro–Wilk test was employed. When both normality and homogeneity of variance were confirmed, a conventional *t*-test was used for statistical analysis. Conversely, if normality was confirmed but homogeneity of variance was not, Welch’s *t*-test, which does not assume equal variances, was applied. In cases where normality was not confirmed, the Wilcoxon signed-rank test was conducted. Statistical significance was defined as a *p*-value less than 0.05. Differentially expressed proteins underwent further functional enrichment analyses through GO and KEGG pathway analyses.

### 2.7. Mass Cytometry

Lung tissues were minced and digested with a solution containing 0.5 mg/mL collagenase I (Yeasen, Shanghai, China) and 0.02 mg/mL DNase I (Roche, Indianapolis, IN, USA). The mixture was then incubated at 37 °C for 45 min, with shaking every 5 min. The digested tissues were homogenized and filtered through 70 µm cell strainers using 5 mL DMEM supplemented with 10% FBS. After centrifugation at 4 °C for 5 min at 400× *g*, the supernatant was discarded. The resulting pellet was reconstituted in 5 mL PBS. A second centrifugation was then performed at room temperature for 20 min at 650× *g*. Red blood cells (RBCs) were lysed using an RBC lysis buffer (Solarbio, Beijing, China). After that, the cells were rinsed with LunaStain cell staining buffer (Polaris Biology, Shanghai, China) and initially treated with Fc block (Biolegend, San Diego, CA, USA) for 10 min at ambient temperature. Cells were incubated with a 10 μL mixture containing Cisplatin and antibodies for 30 min. The antibody panels were detailed in the Supplementary Table S1. They were then washed, fixed with LunaFix for 5 min, washed again, and resuspended in LunaPerm for 30 min. After washing, cells were treated with intracellular antibodies for 1 h, followed by two washing steps. Subsequently, cells were stained with Ir-DNA for 10 min and adjusted to 1 million cells/mL in LunaAcq solution. Cells were acquired using a Lunarion X1 MD mass cytometer (Polaris Biology, China).

After data acquisition, the mass cytometry data were converted into standardized FCS 3.0 file formats using Lunarion 0.92 software (Polaris Biology, China). Manual gating procedures were performed using FlowJo 10.10 software (BD Biosciences, Franklin Lakes, NJ, USA). To obtain a detailed visualization of the immune cell compartment, Uniform Manifold Approximation and Projection (UMAP) dimensionality reduction was applied. Furthermore, to identify distinct immune cell subtypes, FlowSOM clustering and subsequent metaclustering analyses were conducted [28].

### 2.8. Statistical Analysis

Statistical analyses were conducted using SPSS/PC+ version 22.0 software, (Chicago, IL, USA). The results were presented as means with their standard deviations (SD). For normally distributed variables that met the assumption of equal variances, an independent two-sample *t*-test was used to assess differences between two independent groups. If the variables were normally distributed but did not satisfy the assumption of equal variances, the Welch *t*-test was employed. Otherwise, for variables that were not normally distributed, the Wilcoxon rank-sum test was used to compare two independent groups (n = 6). The criterion for statistical significance was set at *p* < 0.05.

## 3. Results

### 3.1. MP Morphology and Concentration

**Figure 1:**
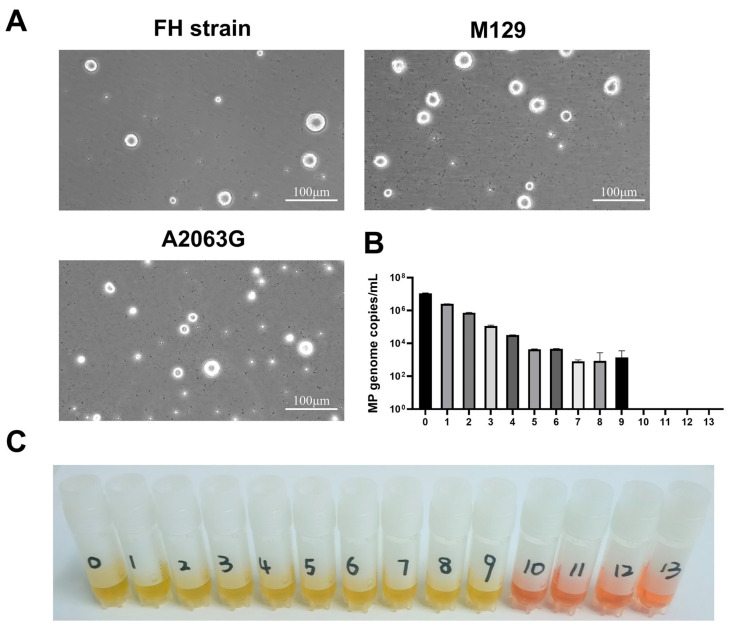
The concentration of *Mycoplasma pneumoniae* was determined to be 10^9^ CCU/mL. (**A**) Morphological characteristics of the standard (FH), M129, and macrolide-resistant (A2063G) strains. (**B**) Real-Time PCR assay for quantification of *Mycoplasma pneumoniae*. (**C**) Quantification of *Mycoplasma pneumoniae* by CCU Assay. In a tenfold serial dilution (10^0^ to 10^13^), the endpoint titer is the highest dilution (10^9^) showing a color change from yellow to red, with 0 to 13 representing the lg values of the dilution factor.

We observed that *Mycoplasma pneumoniae* formed colonies with a thick, dense central core that sinks slightly into the culture medium, surrounded by a thin, translucent layer resembling a “sunny-side-up egg” (Figure 1A). The highest dilution at which color change occurred with detectable MP genome copies was determined and defined as containing 1 CCU/mL. The cultured MP concentration was 10^9^ CCU/mL (Figure 1B,C).

### 3.2. MPP Mice Exhibit Severe Inflammatory Responses and Lung Tissue Damage During the Acute Phase

**Figure 2:**
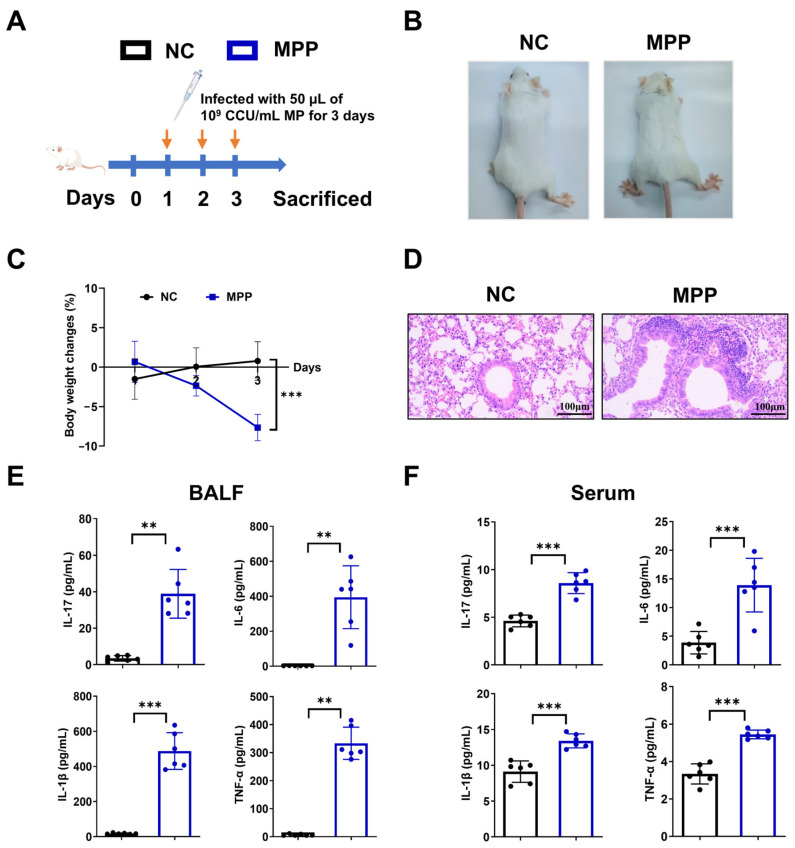
MPP mice exhibited pathological lung tissue damage and elevated levels of inflammatory cytokines (n = 6/group). (**A**) Schemes showing the establishment of MPP mice model. (**B**) General status observation. (**C**) Mice body weight changes. (**D**) HE staining of lung tissues, 20×, Scale bar = 100 μm. (**E**) Kits to evaluate IL-17, IL-1β, IL-6, and TNF-α levels in BALF. (**F**) Kits to evaluate IL-17, IL-1β, IL-6, and TNF-α levels in serum. Data are shown as the mean ± SD, ** *p* < 0.01, *** *p* < 0.001.

To determine the mechanism underlying *Mycoplasma pneumoniae* infection, we established MPP mice model by intranasal challenge with 50 µL of 1 × 10^9^ CCU/mL the standard FH strains, administered for 3 consecutive days (Figure 2A). The results demonstrated that the mice in the Normal group (NC) were in good spirits and actively engaged in activities. They displayed shiny fur without any dullness, and the rate of weight gain increased. In contrast, mice in the MPP group were in poor spirits, appeared fatigued during activities, had visibly ruffled fur and weight loss (Figure 2B,C). Pathological analysis revealed pronounced infiltration of a large number of inflammatory cells and severe tissue damage—including thickening, congestion, and variation in alveolar sizes—in the MPP group compared to NC mice post-infection. This was accompanied by extensive infiltration of lymphocytes, neutrophils, and macrophages (Figure 2D). Meanwhile, the levels of inflammatory factors, IL-17, IL-6, IL-1β, and TNF-α in the BALF and serum samples from the MPP model group were significantly higher than those in the normal group (Figure 2E,F).

### 3.3. Dynamic Changes of Immune Cells in Lung Tissue During Acute M. pneumoniae Infection

**Figure 3:**
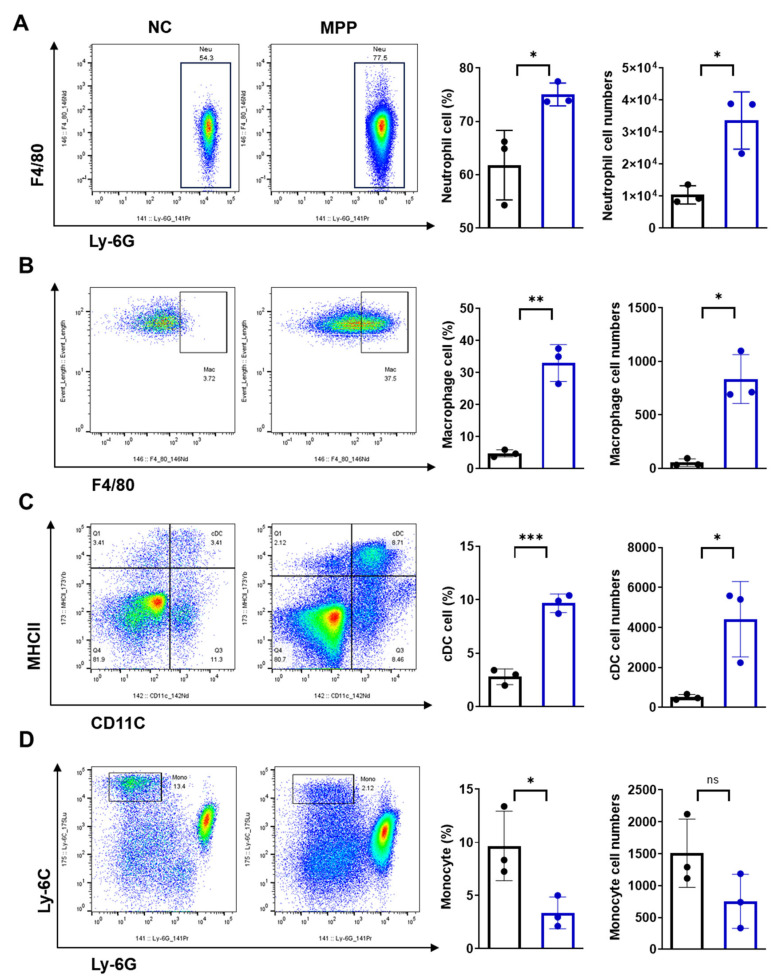
Immune response activation in lung tissue of MPP mice during the acute infection phase (n = 3/group). (**A**) Percentage and absolute cell numbers of neutrophils (Ly-6G+ F4/80−) in lung tissue. (**B**) Percentage and absolute cell numbers of macrophages (F4/80+) in lung tissue. (**C**) Percentage and absolute cell numbers of cDCs (CD11C+ MHC-II+) in lung tissue. (**D**) Percentage and absolute cell numbers of monocyte cells (Ly-6C+ Ly-6G−) in lung tissue. Data are shown as the mean ± SD, * *p* < 0.05, ** *p* < 0.01, *** *p* < 0.001. ns indicates no significance.

*M. pneumoniae* infection can cause disorders of innate and adaptive immunity in the host. The results indicated that the percentages and absolute cell numbers of neutrophils (Ly-6G+ F4/80−), macrophages (F4/80+), and conventional dendritic cells (cDCs) (CD11c+ MHC-II+) in the lung tissue were higher in the MPP group than in the normal group during the acute phase (Figure 3A–C). During the initial phase of MP infection, the immune response was activated. Neutrophils can directly eliminate pathogens through phagocytosis. They also release inflammatory mediators, such as cytokines and chemokines, to amplify the inflammatory response. Subsequently, macrophages are recruited to the infection site to phagocytose pathogens and clear damaged cells, thereby triggering the adaptive immune response. Elevated numbers of cDCs indicate that the body is enhancing its antigen-presenting capacity to activate T cells and B cells, thus promoting a specific immune response. While the percentage of monocytes (Ly-6C+, Ly-6G−) significantly decreased in the MPP group compared to the normal group. The cell numbers of monocytes in the MPP group showed no significant difference compared to the NC group (Figure 3D). Thus, immune responses mediated by neutrophils, macrophages, cDCs, and monocytes may play an important role in the pathogenesis of MPP during the acute phase.

### 3.4. Myeloid Cells Are the Predominant Immune Cells in the Lung Tissue of MPP Mice

**Figure 4:**
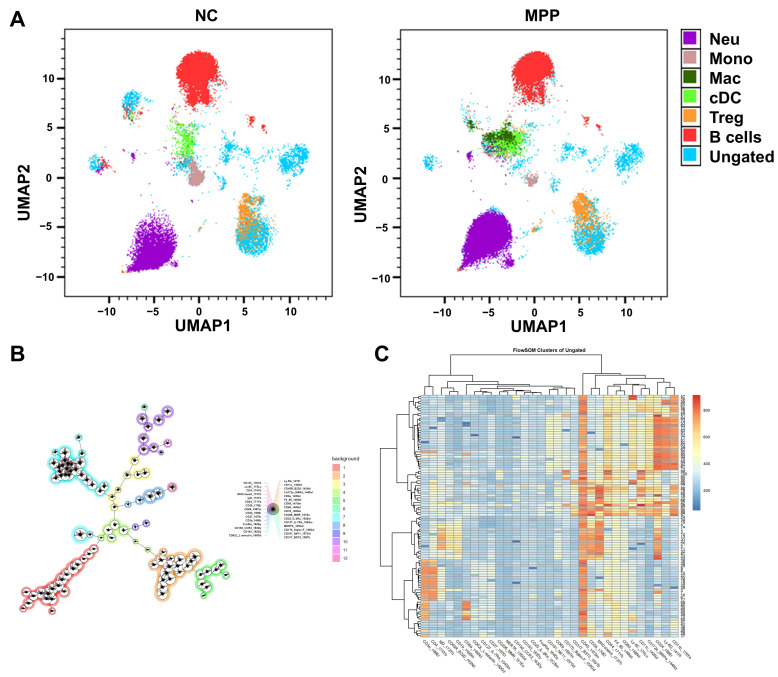
The immunocellular landscape within mice lung tissue. (**A**) The UMAP plot displays the distribution of cells along the UMAP1 and UMAP2 dimensions, with points color-coded to represent major immune cell subpopulations (Purple: Neu; Brown: Mono; Dark green: Mac; Light green: cDC; Orange: Treg; Red: B cell). (**B**) The FlowSOM star diagram illustrates immune cell clusters in lung tissue. Each node represents a FlowSOM cluster, with node size reflecting cluster abundance. Connecting lines indicate similarity between clusters based on marker expression distances. Node labels show major surface markers, including CD11b, Ly6C, CD4, CD8, MERTK, and CD206. The colour scale on the right corresponds to inferred cell types, ranging from red (B cells) to purple (Mac3 macrophage subtype), and includes Treg, cDC, NK, CD8+ T, Neu1/2, cDC2, Mono, Mac1/2. This star diagram reveals the topological structure of the cellular panorama, highlighting the separation of myeloid (left branch) and lymphoid (right branch) clusters. (**C**) Ungated cell FlowSOM-derived clustering heatmap in lung tissue samples compares the MPP group and NC group. Rows correspond to individual cell clusters identified by FlowSOM, hierarchically clustered and visualized via the dendrogram on the left. Columns represent key immune surface markers (e.g., CD3, CD4, CD8, CD19, CD11b, CD14, Ly6C, Ly6G). Colour scales denote median expression levels of metal-labelled antibodies, ranging from low (blue, approximately 200 intensity units) to high (red, approximately 800 intensity units). The top dendrogram groups markers by expression similarity, providing a visual summary of marker relationships.

To further visualize the immunocellular landscape within mice lung tissue, the UMAP algorithm was employed to perform dimensionality reduction on ungated cell data. Subsequently, the proportions of major subpopulations were quantified. Within the MPP group, the UMAP plot revealed a more dispersed cellular distribution, with significant expansion of the purple Neu (neutrophil) and dark green Mac (macrophage) clusters, indicating that the MPP mice induced enhanced neutrophil and macrophage infiltration. Notably, the Neu subpopulation constituted 77.4% of the parent population in the MPP group, markedly exceeding the control group’s proportion of 51.8%. However, their relative proportions within the total cell population were 4.85% and 1.93%, respectively, suggesting a redistribution of inflammatory cell proportions within the MPP group. In contrast, the normal group’s UMAP plot showed a more compact red cluster representing B-cells, with a B-cell proportion of 4.18% (Frequency of Parent). The MPP group’s B-cell proportion was 3.78%, with no significant difference observed between the two groups. Other subpopulations, such as monocytes and cDCs, exhibited lower proportions in the MPP group (0.07% and 0.47%, respectively) (Figure 4A). The observed higher proportions of pneumonia-associated myeloid cells align with this finding. Although the sample size limits definitive functional conclusions, the transcriptional profile of these clusters suggests activated states based on gene expression signatures indicative of phagocytosis and cytokine production. These findings warrant further investigation in larger cohorts.

To elucidate the spatial relationships of immune cell clusters within mouse lung tissue, the FlowSOM algorithm was employed to generate a minimum spanning tree (MST) star diagram, integrating data from both the MPP and NC groups. The primary branch on the left of the figure displays densely clustered myeloid nodes, such as Neu1/2 and Mac1/2/3, which are dominated by CD11b and Ly6C and coloured from blue to purple. These nodes exhibit higher abundance in the MPP group, reflecting MP-induced inflammatory cell recruitment. In contrast, the right branch concentrates lymphoid cells (e.g., Treg, CD8+ T, and B cells expressing CD25, CD8, and CD19, coloured green to red), which exhibit a more balanced distribution in the NC group. However, B cell nodes shrink in the MPP group, suggesting suppression of adaptive immunity. Central nodes (e.g., cDCs and NK cells, coloured yellow-green) connect both branches, indicating transitional subpopulations. These spatial and topological features were examined by cluster similarity analysis, revealing significant differences in node abundance between groups, further providing preliminary support for the concept of myeloid-dominant immune remodeling in the pneumonia model (Figure 4B). The heatmap reflects distinct clustering patterns of disease-induced alterations. Clusters in the lower left region display populations with elevated CD3 and CD8 expression (deep red), indicating cytotoxic T cells; these were more prominent in the MPP group than in the NC group. The central cluster shows increased expression of CD11b, Ly6G, and MHC class II (orange-red), suggesting enhanced neutrophil and cDC infiltration in MPP samples. This is consistent with the inflammatory response in the MPP model. In contrast, B-cell-associated clusters with high CD19 and CD20 expression located at the bottom right exhibited similar expression levels across groups. These findings preliminary reveals that altered expression primarily occurs within myeloid cells in the MPP group, providing offers initial clues into pneumonia-associated immune dynamics (Figure 4C).

### 3.5. The Dysregulation of Cytokines Underlies the Pathogenesis of M. pneumoniae Infection

**Figure 5:**
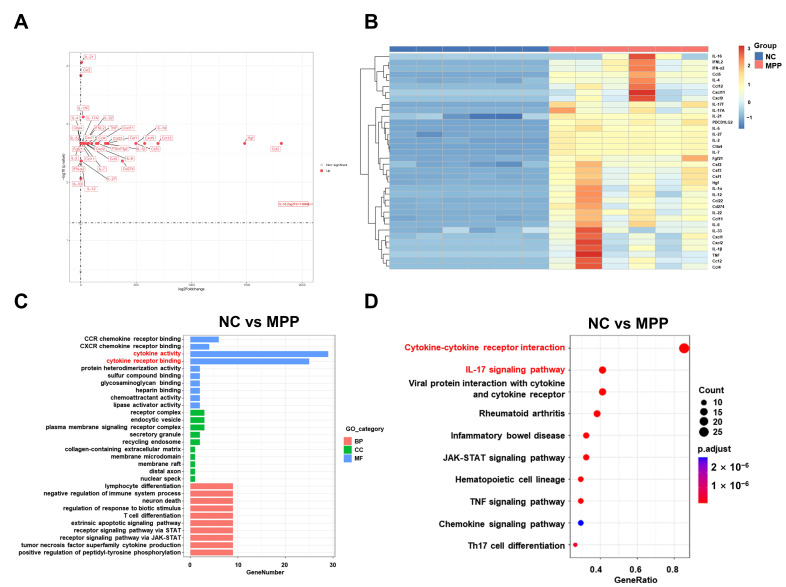
The analysis of cytokines in the acute phase of infection. (**A**) Volcano plots show upregulated protein expression for NC vs. MPP groups in BALF. Each individual data point represents a specific protein. The *x*-axis denotes the protein’s log2 fold change. The *y*-axis indicates the statistical significance, shown as the -log10 of the *p*-value. Proteins positioned on the right side of the graph indicate increased abundance in the samples from the specified group. (**B**) Heatmap illustrates BALF protein expression levels in groups NC and MPP, with blue indicating lower expression and red indicating higher expression. (**C**) The GO enrichment analysis includes three categories: Biological Process (BP), Cellular Component (CC), and Molecular Function (MF). (**D**) KEGG pathway enrichment analysis in NC compared to MPP. The analysis identifies enriched pathways, providing insights into the biological processes involved. The enriched top two pathways are highlighted in red.

At the acute phase of the disease, a total of 36 proteins were significantly upregulated in the BALF of the MPP group. These upregulated proteins include chemokines (Ccl2, Ccl4, Ccl5, Ccl11, Ccl12, Ccl22, Cxcl1, Cxcl2, Cxcl9, Cxcl11), interleukins (IL-1α, IL-1β, IL-2, IL-4, IL-5, IL-6, IL-7, IL-9, IL-10, IL-12, IL-16, IL-17A, IL-17F, IL-21, IL-22, IL-27, IL-33), Csf1, Csf2, Csf3, IFN-α2, IFNL2, TNF, CD274, Fgf21, Hgf, PDCD1LG2, and Ctla4 (Figure 5A). The cellular components, including metabolites and toxins, released by *M. pneumoniae*, could act as pro-inflammatory molecules to stimulate the inflammatory response. This effect is indicated by the upregulation of top 5 most prominent pro-inflammatory cytokines (IL-16, Ccl5, IL-17F, IL-17A, and csf2) in BALF of the MPP mice (Figure 5B). GO enrichment analysis revealed enrichment of biological process terms related to cytokine activity, cytokine receptor binding, and binding to CCR chemokine receptors in the NC versus the MPP group. The main cellular components included receptor complexes, endocytic vesicles, and plasma membrane signaling receptor complexes. In addition, the biological process category included lymphocyte differentiation and negative regulation of immune system processes (Figure 5C). The KEGG pathway analysis revealed significant enrichment of the cytokine–cytokine receptor interaction pathway and the IL-17 signaling pathway in the NC versus the MPP group (Figure 5D).

## 4. Discussion

*Mycoplasma pneumoniae* pneumonia is a significant respiratory infection, primarily affecting children and young adults [29,30]. The disease is caused by the pathogenic bacterium *Mycoplasma pneumoniae*, which is known for the absence of a cell wall, a feature unique among bacteria, making it resistant to many common antibiotics [31]. MPP often presents with symptoms ranging from mild respiratory distress to severe pneumonia. In some cases, it can lead to complications such as acute respiratory distress syndrome (ARDS) and, in extreme instances, death [32,33]. Although MPP typically has a self-limiting nature, severe cases have been documented. This highlights the necessity for further research into the underlying immunological mechanisms and pathogenic pathways associated with this infection.

This study aims to elucidate the immunological responses and cytokine dynamics during acute *M. pneumoniae* infection using a murine model of MPP. We investigate the infiltration of immune cells and the upregulation of pro-inflammatory cytokines to better understand the role of the innate immune system in the pathogenesis of MPP. Our findings reveal a significant increase in neutrophils and macrophages. Additionally, we observe elevated levels of specific cytokines, including IL-17, IL-6, IL-1β, and TNF-α, which collectively contribute to inflammation and tissue damage in the lungs during infection. These insights not only enhance our understanding of MPP but also pave the way for potential therapeutic interventions targeting these inflammatory pathways. Cytokine upregulation and immune cell infiltration are triggered by environmental and host factors, lipoproteins and TLR2 have been thought to be important for the pathogenesis of M. pneumoniae [9]. This recognition activates signaling pathways that produce pro-inflammatory cytokines. The response is further amplified by damage-associated molecular patterns (DAMPs) released from tissue damage, which activate immune cells via the NLRP3 inflammasome, thereby creating a feedback loop that enhances inflammation [34,35]. The IL-23/IL-17 axis, with elevated IL-17 produced by CD4+ T cell subsets, induces specific CXCL chemokines that drive neutrophil infiltration, linking the cytokine storm to the cellular landscape of MPP [15].

The investigation into the immunological mechanisms underlying acute *Mycoplasma pneumoniae* pneumonia revealed significant insights into the molecular pathways activated during infection [36]. We observed a pronounced increase in neutrophil and macrophage infiltration within lung tissues, highlighting the crucial roles of these innate immune cells in responding to *M. pneumoniae* infection. This myeloid dominance could result from either enhanced recruitment of cells from the circulation or local proliferation within the lung microenvironment, a distinction that future studies employing dynamic tracking of cell migration and proliferation will help clarify. Specifically, the dominance of neutrophils, which constituted a substantial percentage of the inflammatory cell population, underscored their dual role in both pathogen clearance and potential tissue damage through cytokine release [37,38]. Moreover, the upregulation of various pro-inflammatory cytokines in BALF, including IL-1β, IL-6, and TNF-α, suggested that these cytokines were pivotal in driving the inflammatory response. This response led to the subsequent lung tissue damage observed in MPP [39,40,41]. These findings contribute to our understanding of the molecular mechanisms at play during M. pneumoniae infections and suggest potential therapeutic targets for controlling the excessive inflammatory response.

Considering genetic and cellular behavior, our findings have significant implications for understanding how immune cells adapt during acute respiratory infections. The observed shifts in immune cell populations, particularly the increase in myeloid cells at the expense of lymphoid cells, indicate a pronounced innate immune response that may suppress adaptive immunity, particularly the functions of B cells [42,43,44]. Our data reveal an association between specific cytokine signaling pathways and the neutrophil-dominated immune landscape observed during MPP. While this correlation suggests a potential modulatory role in neutrophil recruitment and activation, future functional studies are needed to establish definitive mechanistic causality. This alteration in immune cell behavior emphasizes the need for further research into the interplay between innate and adaptive immunity in respiratory infections, as such research may provide novel insights into how the immune system orchestrates responses to pathogenic threats.

The implications of our findings extend into the realm of immunological mechanisms, particularly regarding the potential for future therapeutic strategies specific cytokine pathways. The significant elevation of cytokines, such as IL-17 and IL-6, in the context of MPP highlights their correlation with inflammatory responses. This correlation suggests that modulation of these pathways warrants investigation as a potential approach managing acute pneumonia caused by *M. pneumoniae* [45]. However, the therapeutic potential of such strategies requires direct experimental validation of their function in preclinical models. For instance, future studies could test whether inhibiting IL-6 signaling may reduce inflammatory damage and improve clinical outcomes in patients with severe pneumonia [46]. Additionally, the identification of myeloid cell expansion as a hallmark of MPP may inform the development of new immunotherapies [47]. Preclinical studies are needed to explore whether therapies aimed at modulating myeloid cell function could enhance host defense while mitigating immune-mediated tissue damage [48,49,50]. Our research has revealed that MPP mice and clinically severe *Mycoplasma pneumoniae* patients exhibit similar pathological features, characterized by elevated inflammatory mediators and infiltration of neutrophils and macrophages in BALF. Overall, these findings underscore the importance of understanding the intricate balance between immune activation and regulation. Future research incorporating functional experiments will be crucial to translate these correlational observations into validated therapeutic strategies for infectious diseases such as MPP.

The limitations of this study must be acknowledged to provide a balanced perspective on the results. Firstly, the relatively small sample size may compromise statistical power, particularly when evaluating rare immune cell subsets. This limitation could obscure meaningful insights into their dynamics during *Mycoplasma pneumoniae* infection. Moreover, the lack of human validation in our observations limits the applicability of our findings to clinical settings and requires further investigation within clinical cohort studies. Finally, potential batch effects arising from the multi-platform data integration, including mass cytometry and qPCR, could introduce technical variability; therefore, caution is warranted when interpreting the results within the context of complex biological systems.

## 5. Conclusions

In conclusion, this study elucidates the intricate immune dynamics and cytokine-driven pathology associated with acute *Mycoplasma pneumoniae* pneumonia. The key findings reveal that MPP infection induces a severe local and systemic inflammatory response, marked by a significant cytokine/chemokine storm and a distinct shift in lung immune cell populations. The observed dominance of innate immune responses, particularly the pronounced infiltration of myeloid cells and the resultant cytokine storm, underscores the critical role of these pathways in disease progression. The identification of key inflammatory pathways, including cytokine–cytokine receptor interaction pathway and IL-17 signaling pathway, offers promising avenues for therapeutic intervention. Among these, the CCL5 pathway emerges as a promising target given its role in neutrophil recruitment and macrophage infiltration. Furthermore, targeting the upstream master regulators, such as IL-6 and TNF-α, that drive the cytokine storm could provide an extended therapeutic window. Future research should prioritize the validation of these findings in human cohorts and explore the potential for immunomodulatory therapies to mitigate disease severity, thereby enhancing our understanding of *M. pneumoniae* pathogenesis and informing clinical management strategies.

## Data Availability

The original contributions presented in this study are included in the article/Supplementary Materials. Further inquiries can be directed to the corresponding authors.

## Supplementary Materials

The following supporting information can be downloaded at: https://www.mdpi.com/article/10.3390/microorganisms14010229/s1, Table S1: The mass cytometry antibody panels.

## Abbreviations

The following abbreviations are used in this manuscript:

MPP	Mycoplasma pneumoniae pneumonia
MP	Mycoplasma pneumoniae
SMPP	severe Mycoplasma pneumoniae pneumonia
IL	interleukin
GMPP	general MPP
NP	necrotizing pneumonia
RMPP	refractory MPP
TLRs	Toll-like receptors
AMs	alveolar macrophages
NF-κB	nuclear factor-κB
CCUs	color change units
qPCR	quantitative real-time PCR
BALF	bronchoalveolar lavage fluid
PEA	Proximity Extension Assay
GO	Gene Ontology
KEGG	Kyoto Encyclopedia of Genes and Genomes
RBCs	Red blood cells
UMAP	Uniform Manifold Approximation and Projection
SDs	standard deviations
NC	Normal group
cDCs	conventional dendritic cells
Neu	neutrophil
Mac	macrophage
MST	minimum spanning tree
BP	Biological Process
CC	Cellular Component
MF	Molecular Function
ARDS	acute respiratory distress syndrome
