# Supplementary material for: Immune Dysregulation and Cytokine Profiling in Acute Mycoplasma pneumoniae Pneumonia
Source: Microorganisms. 2026 Jan 19;14(1):229. doi: 10.3390/microorganisms14010229 (PMC12844252; doi:10.3390/microorganisms14010229)
Supplement: Supplementary file 1 [file microorganisms-14-00229-s001.zip › microorganisms-4097248-supplementary.pdf]

Supplementary Table S1 Mass cytometry antibody panels

Antibodies	Clone Numbers	Metal labels
Ly-6G	1A8	141Pr
CD11c	N418	142Nd
CD45R/B220	RA3-6B2	143Nd
CD172α/SiRPα	P84	144Nd
CD8α	53-6.7	145Nd
F4/80	BM8	146Nd
CD45	30-F11	147Sm
CD80	16-10A1	148Nd
CD19	6D5	149Sm
CD206/MMR	C068C2	151Eu
CD25/IL-2Rα	PC61	152Sm
CD127/IL-7Rα	A7R34	154Sm
MERTK	2810C42	155Gd
CD170/Siglec-F	S17007L	156Gd
CD161/NK1.1	QA19A51	157Gd
CD317/BST2	927	159Tb
CD62L/L-selectin	MEL-14	160Gd
CD163	S15049I	162Dy
CD192/CCR2	QA18A56	163Dy
FccRlα	1-Mar	164Dy
CD3e	17A2	166Er
CD27	LG.3A10	167Er
CD24	M1/69	168Er

CD69	H1.2F3	169Tm
CD38	90	170Er
CD44	IM7	171Yb
IgD	11-26c.2α	172Yb
MHC class II	M5/114.15.2	173Yb
CD4	RM4-5	174Yb
Ly-6C	HK1.4	175Lu
CD11b	M1/70	176Yb
